# Parental decision-making following a prenatal diagnosis that is lethal, life-limiting, or has long term implications for the future child and family: a meta-synthesis of qualitative literature

**DOI:** 10.1186/s12910-019-0393-7

**Published:** 2019-08-08

**Authors:** Claire Blakeley, Debbie M. Smith, Edward D. Johnstone, Anja Wittkowski

**Affiliations:** 1 The University of Manchester, Faculty of Biology, Medicine and Health, School of Health Sciences, Division of Psychology and Mental Health, 2nd Floor, Zochonis Building, Brunswick Street, Manchester, M13 9PL UK; 2 Greater Manchester Mental Health Foundation Trust, Trust Headquarters, Bury New Road, Prestwich, Manchester M25 3BL UK; 3 Leeds Trinity University, Brownberrie Lane, Horsforth, LS18 5HD Leeds UK; 4 Central Manchester University NHS Foundation Trust, Oxford Road, Manchester, M13 9WL UK

**Keywords:** Termination, Continuation, Pregnancy, Life-limiting, Abortion, Birth, Disorder

## Abstract

**Background:**

Information on the factors influencing parents’ decision-making process following a lethal, life-limiting or severely debilitating prenatal diagnosis remains deficient. A comprehensive systematic review and meta-synthesis was conducted to explore the influencing factors for parents considering termination or continuation of pregnancy following identification of lethal, life-limiting or severely debilitating fetal abnormalities.

**Methods:**

Electronic searches of 13 databases were conducted. These searches were supplemented by hand-searching Google Scholar and bibliographies and citation tracing. Thomas and Harden’s (2008) thematic synthesis method was used to synthesise data from identified studies.

**Results:**

Twenty-four papers were identified and reviewed, but two papers were removed following quality assessment. Three main themes were identified through systematic synthesis. Theme 1, entitled ‘all life is precious’, described parents’ perception of the importance of the fetus’ life, a fatalistic view of their situation alongside moral implications as well as the implications decisions would have on their own life, in consideration of previous life experiences. Theme 2 (‘hope for a positive outcome’) contained two sub-themes which considered the parent’s own imagined future and the influence of other people’s experiences. Finally, Theme 3 (‘a life worth living’) presented three sub-themes which may influence their parental decision-making: These described parental consideration of the quality of life for their unborn child, the possibility of waiting to try for another pregnancy, and their own responsibilities and commitments.

**Conclusion:**

The first review to fully explore parental decision-making process following lethal, life-limiting, or severely debilitating prenatal diagnosis provided novel findings and insight into which factors influenced parents’ decision-making process. This comprehensive and systematic review provides greater understanding of the factors influential on decision-making, such as hope, morality and potential implications on their own and other’s quality of life, will enable professionals to facilitate supported decision-making, including greater knowledge of the variables likely to influence parental choices.

## Plain English summary

Little is known about how parents make choices after finding out their unborn child has a disorder or illness which might seriously affect their chance of surviving or increase their likelihood of having a severe disability after birth. For this reason, we undertook a review of studies that help us understand how parents make decisions.

To perform our analysis we searched 13 different databases, Google Scholar and reference lists. We chose to include only qualitative studies in our review. We reviewed papers resulting from these studies using a method described by Thomas and Harden (2008). The search identified 24 papers which discussed how parents make decisions after being told their unborn baby had a serious illness but two papers were removed because they were poor. The final 22 papers revealed that parents had many thoughts about whether to carry on with or end their pregnancy. These thoughts included how special the pregnancy was to them. They were also hopeful that their pregnancy would end well. Finally, they thought about whether their baby would suffer if he or she survived.

The findings of this review will be helpful for health professionals who look after women and their partners when problems are identified during pregnancy. We offer new insights into why and how parents reach decisions they do about what course of action they take.

## Introduction

The identification of lethal, life-limiting, or severely debilitating fetal abnormalities prenatally has increased over the past few decades, with prenatal screening standard practice in many countries [1]. Approximately 2–3% of all parents attending prenatal clinics for screening receive results suggestive of fetal abnormality [2, 3], leaving them with emotional decisions to make in relation to the continuation or termination of pregnancies [4, 5]. Between 81 and 90% of parents terminate pregnancies identified with lethal, life-limiting, or severely debilitating disorders [6–8], whilst others choose to continue with their pregnancy. However, no concise review detailing all factors influencing parental decision-making when parents are faced with the option to continue or terminate a pregnancy affected by lethal, life-limiting, or severely debilitating prenatal diagnosis has been provided; hence there is a need for further clarification to ensure adequate support is provided during this period.

Parents deciding to continue or terminate a pregnancy following diagnosis of fetal abnormality experience grief, shock [9–11], disbelief, isolation, anger and adaptation or adjustment [9, 10, 12]. Studies have identified several influential factors on parental decision-making following prenatal diagnosis. Expectant parents’ observation of ultrasound examinations influenced their attachment to the fetus [13, 14] and a diagnosis of fetal disorder was not found to disrupt this attachment [15]. Advanced maternal age is a contributing factor in attachment and personification of a fetus, especially for women who perceive the pregnancy to be their final chance to give birth [16], with this attachment increasing through the development of the pregnancy [17, 18]. Religion has also been suggested as a leading factor in decision-making for parents faced with the option to terminate a fetus [18–20]. For example, faith has been found to encourage hopeful and fatalistic beliefs [21], with fear of disobeying religious scripture playing an important role in decision-making [22].

Whilst studies provide examples of factors influencing parental decision-making, they do not infer how maternal healthcare teams working with parents should best support them in making decisions beyond non-judgemental support [23] and counselling for likely outcomes [24], leaving important recommendations to better prepare maternal healthcare teams for this supportive role. The studies discussed were largely quantitative in methodology [16–20], with qualitative studies focussing on hypothetical scenarios and cultural acceptance of termination [18, 22] or the experience of mothers in countries where termination is illegal [21]. An exploration of parents’ lived experiences following prenatal diagnosis of lethal, life-limiting, or severely debilitating diagnosis provides insight into the thoughts of parents in this unique situation, informing the evidence base of influential factors in parental decision-making, and how maternal healthcare teams can facilitate and support parents during this time.

Within their syntheses of parental experiences of prenatal diagnosis, three reviews provided an insight into parental experiences at the time of making a decision [25–27]. In their review of 17 studies, Sandelowski and Barrosso [25] investigated the experience of diagnosis of any prenatal condition following screening for expectant parents in the USA. The authors highlighted some influential factors in decision-making: Parents reported that they combined new information with prior knowledge or beliefs about disability, parenthood and the moral or religious acceptability of abortion when making their decision to terminate or continue an affected pregnancy. Parents considered the certainty of the diagnosis, their ability to parent a child with the disorder and how this would impact others, their prior fertility, support from family and friends, and the pressured timeframe within which to make their decision. Parents also discussed their anticipated hope that their child would be born without the disorder and their perceived guilt around not allowing their pregnancy to continue. This 2005 meta-synthesis was limited in its sample of 17 studies, due to geographical and language biases. The synthesis also included 10 studies, which were unpublished at the time of the review, with risk of reduced quality.

Lafarge et al.’s meta-ethnography [26] reviewed 14 English language studies which addressed women’s experiences of choosing to terminate a pregnancy, following prenatal diagnosis. They identified that women expressed feelings of guilt and hopelessness when deciding to end their pregnancy. However, they also considered the baby’s potential quality of life as well as the impact on them and their wider family. The review focussed on the women’s experience of the termination process, addressing women’s decision-making in two sub-themes, whilst the remaining ten sub-themes focussed on the experience of women. Studies were not excluded from this review if they did not consider parental decision-making.

Lou et al’s [27] qualitative systematic review presented findings on parents’ experiences of prenatal diagnosis, discussing their relationship with clinicians, other’s acknowledgment of their pregnancy and stigmatization experienced. This review of 28 studies of parents within European or English speaking countries highlighted the multiple losses experienced by parents during this difficult time, but it did not address the decision-making process parents are faced with following prenatal diagnosis.

These reviews did not provide a breadth of understanding in relation to factors influencing decision-making in the period following prenatal diagnosis of a lethal, life-limiting, or severely debilitating condition. In order to guide clinical practice in the support of parents following prenatal diagnosis of lethal, life-limiting, or severely debilitating disorders, more information is required about the decision-making process. Studies have examined decision-making as a psychological construct, with normative models, such as Classical Decision-Making Theory [28], aiming to rationalise a complex and individual process [29]. Whilst this model provides insight into how best to approach a decision, it does not necessarily describe how people choose in real-life situations. Descriptive models, such as Prospect Theory [29], provide insight into how individuals make real-life choices. Descriptive models suggest that schemata, encompassing prior life experiences, are drawn on to make decisions [30], considering potential outcomes of each option available to them, and the likelihood of each option occurring [30]. Accoding to Emotion-Imbued Choice Theory [31], individuals imagine their perceived emotional response to each available outcome as a way of mediating decisions. Individually salient impacting factors, such as culture [32], current emotional state [33], delivery of information [34] and time constraints [35], must be considered when supporting parents making decisions.

Thus, the aim of this review was to explore parental decision-making processes following diagnosis of a lethal, life-limiting, or severely debilitating prenatal diagnosis systematically and highlight areas of recommendation for current maternal healthcare practice.

## Methodology

This systematic review and meta-synthesis of qualitative studies followed PRISMA guidelines [36]. The following databases were searched: Allied & Complementary Medicine (AMED), EBM Reviews, Econlit, Embase, Global Health Archive, Health and Psychosocial Instruments, Health Management Information Consortium (HMIC), International Pharmaceutical Abstracts, Maternity and Infant Care Database, MEDLINE, The Philosopher’s Index, PsychINFO, and Social Policy and Practice. The search was inclusive of all years, retrieving papers from the inception of each database up until 27 October 2017. Electronic database searches were supplemented by individual searches of recent editions of relevant journals as well as Google scholar. The review aimed at being inclusive of all papers meeting the inclusion and exclusion criteria outlined in Table 1.

**Table 1:** Inclusion and exclusion criteria

Inclusion criteria	Exclusion criteria
Parents who experienced prenatal diagnosis of lethal, life-limiting, or severely debilitating disorder following prenatal screening	
Studies examining or considering in any manner parents’ decision-making and the factors influencing their decision following the diagnosis of a lethal, life-limiting, or severely debilitating disorder, prior to termination or birth	Decisions relating to: Pre-implantation genetic screening Acceptance of screening Type of termination Choices after termination of pregnancy Choices following birth of child
Studies which used qualitative methods for data collection and analysis	Studies which captured only quantitative data or used quantitative analysis
Studies written in any language	
Primary research	Book reviews, opinion pieces, conference posters or abstracts, literature reviews.

**Fig. 1:**
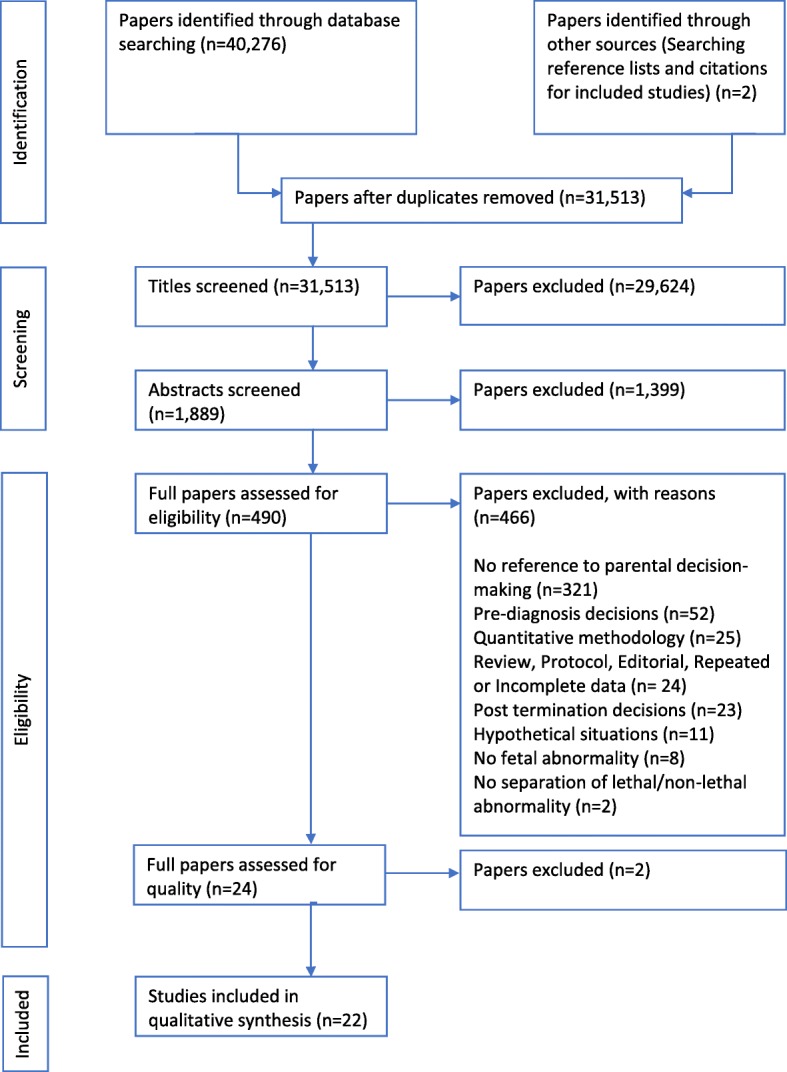
PRISMA flow diagram, representing the study selection process

The systematic search process is illustrated in Fig. 1. No new articles were highlighted for inclusion when the search was updated on 22^n^ June 2018.

A check of 10% of titles, abstracts and full papers reviewed in the search process was conducted by an independent researcher.

### Critical appraisal

All included papers were assessed for quality using the widely used Critical Appraisal Skills Programme (CASP) checklist [37]. As this checklist does not propose a scoring system for which to interpret the quality of each study, the Health Evidence Checklist scoring was also used [38].

### Meta-synthesis

The thematic synthesis approach described by Thomas and Harden [39] was utilised to identify themes presented within the included studies. Building on the concept of thematic analysis, this approach allows for results from different methods of qualitative analysis to be synthesised [40]. The synthesis was implemented in three stages of analysis described by Thomas and Harden [39]. Stage 1 of the process involved both the first author (CB) and second author (DMS) reading each of the included studies a number of times, before independently implementing line-by-line coding within the texts of the results or findings sections of each paper in order to develop codes describing the meaning or content. This process was repeated for each included study, enabling the translation of concepts between the studies as well as identification of new themes. This approach enabled a higher level of analysis to be conducted at stage 2, when both researchers grouped together codes to form descriptive themes apparent across and between papers. The third and final analytical stage of interpreting consistent and inconsistent themes within papers enabled the presentation of analytical themes and sub-themes. This final stage relied on researcher inference and judgement regarding the meaning behind each code. To ensure reliability, both researchers coded and grouped themes independently, before discussing their views with each other and forming the final themes which were then discussed with and agreed by all authors.

## Results

### Quality rating

The majority (*n* = 19) of the initial 24 included studies were judged to be of low bias and high methodological quality. Three studies [41–43] were rated to be of acceptable quality, which indicated these studies had a moderate risk of bias. Two studies [44, 45] were excluded from this review due to risks of low quality and high methodological bias, leaving a final 22 papers for the synthesis. Studies are presented in Table 2, grouped by the decision-making of parents who continued pregnancies, terminated pregnancies or explored both choices.

**Table 2:** Characteristics of included studies presented as studies exploring experiences of parents who continued pregnancies, terminated pregnancies, and studies exploring both

	Study	Research Aims	Sample size & Diagnosis	Participant Characteristics	Data Collection	Methodology & Analysis	Themes	CASP Rating (Cate-gory)
Studies describing pregnancy continuation								
1	Helm et al. [46] 1998 USA	To explore the experience of mothers who continued a pregnancy following diagnosis of Down Syndrome (DS)	Ten mothers who had received a prenatal diagnosis of DS	Six Catholic, three Protestant, one Jewish All European-American Aged 28–42 Nine living with husband, one single	Recruited through a University affiliated centre at local hospitals. Mothers expressed interest in the study before being contacted. Semi-structured, open-ended interviews were then conducted over 2–4 h	Thematic analysis with approximately 10% of interview content used	Religion Morality Personal experience with people with disabilities. A Previous infertility Family Friends Religious leaders Clinicians Genetic counsellors	8 (A)
2	Redlinger-Grosse et al. [47] 2002 USA	To explore parents’ experiences in deciding to continue a pregnancy, given the prenatal diagnosis Holoprosen-cephaly (HPE)	Twenty-four parents who had received a prenatal diagnosis of HPE	Fourteen women and 10 men Aged 23–50 Twenty-one white, 2 Hispanic and 1 Asian Twenty-two married, 1 single and 1 separated or divorced. Twenty-three had living child, 1 had no living children Thirteen Catholic, 7 Protestant and 4 Baptist Twelve completed high school, 6 college, 5 graduate degree and 1 post-graduate degree Fifteen were employed full time, 7 unemployed and 2 worked part-time	Participants were recruited from a conference, research centre, university and support services for families affected by HPE during 2000. In-person interviews were conducted for 8 parents attending the conference, with telephone interviews for 16 parents recruited through the remaining sites, using open-ended questions	Thematic analysis [48]	Religion Fatalism Morality Valuing mother role Previous infertility Prior pregnancy termination Anticipated guilt Personal connection to HPE	8 (A)
3	Rempel et al. [49] 2004 USA	To explore parents’ decision-making and interactions with health care professionals during the antenatal diagnosis of Coronary Heart Diseases (CHD)	Thirty-four parents who had received a prenatal diagnosis of CHD	Nineteen mothers and 15 fathers All intended to continue with the pregnancy Over half first-time parents Gestational age when diagnosed was 18–36 weeks	In-depth, open-ended interviews during the pregnancy, 1 month after the baby’s birth and between 4 and 6 months after birth. The interviews ceased following data saturation	Symbolic interactionism guided analysis [50]	Search for information, Impact on themselves Impact on wider family Health care professionals Attachment	8 (A)
4	Hickerton et al. [51] 2012 Australia	To explore parents’ experience of continuing a pregnancy where a genetic condition was detected prenatally	Nine parents	Five mothers and 4 fathers of children < 10 years old with life-limiting disorders All had prenatal diagnosis, or were warned of high reproductive risk Four skeletal disorders, 2 other organ difficulties, 2 Trisomy syndromes and 1 other chromosomal disorder All fluent in English All married and lived together	Purposive sampling via advertisement in the newsletter of a genetic support network, or through receiving a letter from staff at genetic clinics Semi-structured face-to-face interviews were conducted with mothers and fathers separately, ranging from .5–.2 h	Grounded Theory using a constant comparative approach [52]	Morality Quality of life	9 (A)
5	Hurford et al. [53] 2013 USA	To explore women’s decisions to continue a pregnancy affected by DS after prenatal diagnosis, and the factors that affected their decision	Fifty-six mothers who had received a prenatal diagnosis of DS	Women aged between 21 and 44 Forty-one Caucasian, 8 Hispanic, 3 Asian, 2 African American, and 1 other, Fifty Christians, 2 Atheist, 1 Muslim,1 Jewish, Forty-four very/somewhat religious, 11 not/not very religious, Thirty-two had a college education, 17 postgraduate qualifications, 5 high school education	An information sheet was sent through respective organizations This information sheet provided the web address for the online survey which included 9 free response questions analysed for this study	Thematic analysis	Attachment Religion Morality Personification of fetus Family Friends Belief fetus is alive Fatalism Previous infertility	8 (A)
6	Guon et al. [54] 2014 Worldwide	To explore decision-making of parents who continued their pregnancy after a pre-natal diagnosis of Trisomy 13–18	One-hundred and twenty-eight parents who had received a prenatal diagnosis of Trisomy 13–18	Thirty men and 98 women who had accessed Facebook and other support sites dedicated to Trisomy 13–18. One hundred and six were from the US, 6 Canada, 6 UK, 9 from 12 other countries Parent’s median age was 38 All completed high school, 94 at least one university degree, 27 also completed postgraduate studies One-hundred and nine were religious, with 73 attending religious services All were parents of children who live(d) with full T13–18, mosaicism, and other structural variations involving chromosomes 13 and 18 Ninety-seven had full T13 or 18 The median age of survivors was 3 years	Participants were recruited on English speaking, online support sites relating to Trisomy 13–18 respondents completed a questionnaire, with 5 open ended questions from this used within the study analysis	Thematic qualitative content analysis	Morality Religion Personal values Attachment Uncertainty Hope Desire to be a parent Desire to meet their child Pressure from others	9 (A)
7	Gesser-Edelburg & Shahbari [55] 2017 Israel	To explore Muslim Arab women’s reasons for continuation of a pregnancy following the detection of a congenital anomaly	Twenty-four mothers who had received a diagnosis of congenital abnormal-ities	Recruited between 2014 and 2015 All married All Muslim Aged between 21 and 39	Five participants were recruited by professionals; the rest were through snowballing Interviews lasted 50–60 min, with recruitment ceased following saturation The questions were open-ended and probes were used to elicit more information	Constructivist classical grounded theory [56]	Religion Fatalism Attachment Pressure	9 (A)
8	Moudi & Miri-Moghaddam [57] 2017 Iran	To explore the reasons women with a pregnancy affected by Beta-Thalassemia (B-TM) continued with pregnancy	Thirty-nine mothers who had received a prenatal diagnosis of BTM	Women who had continued with pregnancy Thirty-three were of Sunni religion and 6 Shiite Muslims Aged between 20 and 34 years Five of the women had previously aborted 1 pregnancy, whilst one woman had aborted 2 or more previous pregnancies	Exploratory qualitative methods after purposive sampling were used to identify potential cases In-depth, semi-structured interviews were conducted between 2012 and 2013, terminating when data saturation was reached Interviews lasted approximately 1 h and used open-ended questions	Grounded theory principles [56]	Belief in accuracy Trust in professionals Lack of understanding Consequences to family Family opinions Future fertility Preference for large family Preference for male fetus Lack of siblings Hope for family with husband Morality Religion Attachment	9 (A)
Studies describing pregnancy termination								
9	Bryar [58] 1997 USA	To explore the experiences of women terminating a pregnancy in the 2nd trimester due to discovery of a fetal abnormality	Three women who had received a diagnosis of severe fetal abnormal-ity	Women living in USA who had attended a clinic for abortion Aged between 30 and 40 years All married, white, middle class graduates	Convenience sampling, approaching all women attending follow-up visit 4 weeks after their abortion, within a single private outpatient perinatal practice Unstructured interviews were completed 4–6 weeks after termination The interviews lasted 75–90 min and asked open questions with additional questions asked for clarification	Phenomenology [59]	Religion Family impact Societal expectations Impact on self Potential future difficulties Seriousness of disorder	9 (A)
10	Ferreira da Costa et al. [60] 2005 Brazil	To explore the experience of women in Brazil during the process of abortion for fetal disorder	Ten women who had received a diagnosis of severe fetal disorder	Women who had terminated a pregnancy following legal authorisation in 2002 Aged 17–29 Two were single All had completed at least primary school education	Interviews were conducted 40 days after termination when the women returned for post-termination check- up and genetic counselling The sample was determined by saturation, with semi-structured interviews following a thematic script	Thematic analysis with the methodology of Minayo [61] was used	Seriousness of disorder Risk to own health Reducing suffering	9 (A)
11	Leichtentri-tt [62] 2011 Israel	To explore the experience of women who undergo feticide in Israel	Thirteen women who had received positive prenatal diagnosis	Aged between 28 and 41 All married, with one woman remarried Five received a prenatal diagnosis of Brain or (CNS) defects, 3 received diagnosis relating to other organ difficulties, 2 heart defects, 1 sex chromosome abnormalities, 1 other chromosome abnormalities and 1 fetal growth restriction	The women were recruited from one of the largest hospitals in Israel, and through snowballing techniques between 2008 and 09 Interviews took place 4–24 months after the termination using in-depth semi-structured interviews lasting approximately 3 h	Thematic analysis using the approach of Gubrium and Holstein [63]	Pressure from professionals Uncertainty Preventing suffering Time pressure Legal requirements Family opinion	9 (A)
12	Benute et al. [64] 2012 Brazil	To explore women’s reasons for termination of pregnancies after ultrasound presented findings consistent with lethal fetal malformation	Two-hundred and forty-nine women who received positive prenatal results	Women recruited between 1998 and 2008 Aged between 18 and 33 years One-hundred and thirty-five received diagnosis of anencephaly, 26 urinary disorders, 24 genetic syndromes, 21 CNS, 21 multiple MF, 13 Column MF, 8 conjoined twins and 1 abdominal wall disorder Fetus death prior to the interview led to exclusion One-hundred and sixty-two were Catholic, 54 Evangelical One-hundred and seventy-seven cohabited with partner, 72 single One-hundred and seventy-two opted for termination, 77 had not requested abortion	The sampling was carried out by exhaustion. A psychologist conducted open interviews immediately after the ultrasound diagnosis, with each interview lasting approximately 1 h	Content Analysis was utilised	Reducing suffering Guilt Morality	8 (A)
13	Gawron et al. [65] 2015 USA	To explore the reasons for termination timing among patients whose pregnancy was effected by fetal abnormalities	Thirty women who had received diagnosis of severe fetal disorders	English speaking, adult women presenting for pregnancy termination between 12 and 24 weeks at a tertiary referral family planning clinic between 2011 and 12 Aged between 26 and 44 All were married or in a relationship All had some college education, 12 post-college qualifications Twenty were employed Sixteen were Christian, 9 Athiest, 3 Jewish, 1 Muslim and 1 Hindi Several women had previously terminated pregnancies, including 3 for fetal abnormalities	Convenience sampling with phone or pre-operative consultation recruitment until data saturation reached The interview occurred on the first of a 2-day termination process, prior to medical team interaction Semi-structured interviews using guide focused on decision-making was used, with follow-up questions lasting up to 60 min	Interviews were analysed using latent grounded theory [56]	Partner Shared decision-making Family Friends Religion Suffering for fetus	8 (A)
14	Ioannou et al. [66] 2015 Australia	To explore the experiences of couples that were both identified as carriers of Cystic Fibrosis (CF), and the reproductive decision-making and psychosocial impact when pregnant	Four parents who had received a prenatal diagnosis of CF	Four Individuals forming 2 couples taken from a larger sample included within the study were screened between 2006 and 2012 and found to be in a ‘carrier couple’ One of the pregnancies had occurred through IVF Both pregnancies were terminated	Open-ended questions, informed by the literature and process of screening, were used in the semi-structured interview schedule	Inductive content analysis [67]	Disbelief Hope Prior decision-making	9 (A)
Studies describing pregnancy termination and continuation								
15	Sandelows-ki & Jones [68] 1996 USA	To explore the experience of couples who learn during pregnancy that their baby has a severe fetal impairment	Twenty-seven parents who had been prenatally diagnosed with a severe fetal disorder	Fifteen women and 12 of their male partners (11 of whom were married couples) Women aged between 19 and 40, men between 22 and 39 Largely Euro-American, except for 1 African-American couple, 1 African-American woman, and 1 Asian-American woman Two had terminated previous pregnancies for reasons other than fetal health Eight women continued their pregnancies, 2 losing their babies within 1 h and 1 week of birth, 5 women terminated pregnancies with live fetuses and 2 women terminated pregnancies after a fetal demise	Forty interviews completed with 10 couples and 2 women who were interviewed 2–5 times, with 2 couples and 1 woman interviewed once All participants were interviewed within 11–60 days of learning of the fetal impairment or termination Five couples terminating pregnancies were subsequently interviewed around their due date and the anniversary date of termination Couples continuing pregnancies were interviewed 1–2 times during pregnancy and 1–4 times after delivery Two women conceived during the study and they were interviewed during a second round of prenatal screening Interviews were conducted in a minimally structured manner, with questions asked only to clarify	Narrative analysis [69]	Reducing own distress Reducing fetus’ distress Religion Attachment Own needs Future needs of fetus Ability to care for child with disability	8 (A)
16	Locock, & Alexander [70] 2006 UK	To explore how men experience fetal screening and diagnosis	Seventeen parents were recruited following positive prenatal screening	Eight had received prenatal diagnosis of chromosomal defects, 5 heart defects, and 4 neural tube defects Parents were interviewed either during pregnancy or within 2 years after birth or termination between 2003 and 2004	Participants were recruited using a maximum variation sample, through a national network of GPs, antenatal clinics and classes, national voluntary associations and support groups Narrative interviews were conducted	Modified grounded theory approach [71]	Legality Exclusion Joint decision-making Role as supporter Focussing on positives Seeking information Hard facts Intuition	9 (A)
17	Balkan et al. [41] 2010 Turkey	To explore factors influencing parental decisions to terminate or continue a pregnancy with chromosomal abnormality diagnosed prenatally	Seventy-six parents were recruited following positive prenatal screening	Thirty-eight couples from South-East Turkey who received positive prenatal diagnosis between 2004 and 07 All were Muslim Thirty-two of the forty receiving a diagnosis of DS terminated, all receiving Trisomy 18 diagnosis terminated, and 8 of the 10 receiving a diagnosis of Turner Syndrome (TS) terminated. None of the 6 parents receiving a diagnosis of Klinfelter Syndrome (KS) terminated, with all 4 parents receiving a diagnosis of 47XXX, 2 receiving marker chromosome abnormality or 4 receiving Trisomy 13 terminating	Semi structured, face-to-face interviews lasting one to several hours	No explanation of exact methodology However, authors report that qualitative analyses were conducted	Religion Seriousness of condition	6 (B)
18	France et al. [72] 2012 UK	To explore the role of women’s and couple’s experiences of disabilities in influencing their decision regarding termination	Twenty-eight parents, who had received positive prenatal diagnosis	Twenty-four women and 4 of their male partners with prior experience of a disability Ten had received a prenatal diagnosis of autosomal disorders, 3 structural disorders, 3 heart defects, 5 blood disorders, 3 multiple disorders and 1 muscular disorder Twenty had terminated and 8 continued with their pregnancy Interviews completed 1–12 years after pregnancy Participants aged 23–52 One participant was Pakistani, 1 Black Sierra Leone, 3 Black Nigerian and 23 White Two were single, 3 cohabiting and 23 married People’s experiential knowledge of disability included having a disorder themselves, living with a disabled sibling, and talking to or observing family, friends, acquaintances or clients who had a disability or a disabled relative	Data was collected as part of larger scale study Interviews were originally conducted by two researchers between 2004 and 06 Narrative interviews with participants from a purposive sample Recruitment via GPs, hospital consultants, nurses, support groups and word of mouth Interviews lasted 1–3 h	Framework analysis influenced by Bury’s [73] and Lawson & Pierson’s [74] frameworks	Shared decisions with partner Other’s experiences of condition Experiential knowledge Their own suffering Suffering of fetus Religion Medical advice Imagined futures	9 (A)
19	Huyard [43] 2012 Belgium, France & Germany	To explore the information parents, whose child has an intellectual disability, considered important when deciding whether to terminate following prenatal diagnosis	Four participa-nts from a larger presented sample, who had received a prenatal diagnosis of a severe fetal disorder	Thirty-three interviews were conducted in Germany, France, and Belgium between 2008 and 10 among women, men, or couples who had at least one child with a life-limiting condition Only 4 participants from this sample had experienced a prenatal diagnosis Of the relevant 4 parents, 3 had continued with the pregnancy, and 1 had terminated	The interviewees were recruited through self-help groups of parents whose children have an intellectual disability, or through professionals working in schools or residential centres for people with intellectual disability Semi-structured interviews were conducted sequentially, and followed a 30-item guide	Classical grounded theory methods [56]	Longing for child Fatalism Ability to cope Morality	6 (B)
20	Hodgson et al. [75] 2016 Australia	To explore social and professional supports utilised by parents at the time of lethal, life-limiting, or severely debilitating fetal diagnosis	One-hundred and two parents who had received a severe fetal diagnosis	Seventy-five women and 27 men, all were English speaking	Purposive, convenience sampling was used Interviews were conducted with couples using a semi-structured guide following a narrative and chronological style	Thematic analysis	Provision of information Attitude of information giver Likely prognosis Termination options Previous views Perceived impact on family Other’s perceptions Other’s experiences	9 (A)
21	Fleming et al. [42] 2016 Switzerland	To explore the experiences of parents following severe fetal diagnosis	Thirty-two parents who had received a severe prenatal diagnosis	Seventeen mothers, 1 father and 7 couples were recruited between 2013 and 14 from the German speaking part of Switzerland All had experienced a lethal fetal diagnosis in the previous 5 years	Participants were recruited through a telephone counselling service available to anyone with interest in perinatal loss Data was collected by semi-structured interviews lasting 40–90 min	Thematic analysis in accordance with the method of Braun and Clarke [76] was utilised	Time pressure Searching for information Financial implications	6 (B)
22	Reed & Berrier [77] (2017) USA	To explore decision-making following prenatal diagnosis of DS	Nine parents who had received a diagnosis of DS	Two couples and 5 mothers recruited between 2012 and 2013 All had received a prenatal diagnosis within three years prior to the interview Four had continued their pregnancy, 3 had terminated and 2 had continued with an adoption plan Aged between 26 and 52 Seven females and 2 males Seven Caucasians, 1 Hispanic, and 1 Bi-racial participant Four participants did not affiliate to any religion, 2 were Catholic, 2 Protestant and 1 Lutheran	A convenience sample was used to recruit participants, with expansive cognitive interviewing utilised Interviews were 90 min in length and directed participants to read and complete the questionnaire by “thinking aloud” whilst “concurrent probing” encouraged participants to further elaborate	Thematic analysis [78]	Pressure from professionals Provision of information Scientific information Confidence in professionals Ability to care for child Family impact Support groups Prior experience with DS Other’s experiences Age Attachment Personal values Anticipated quality of life	8 (A)

### Themes

**Fig. 2:**
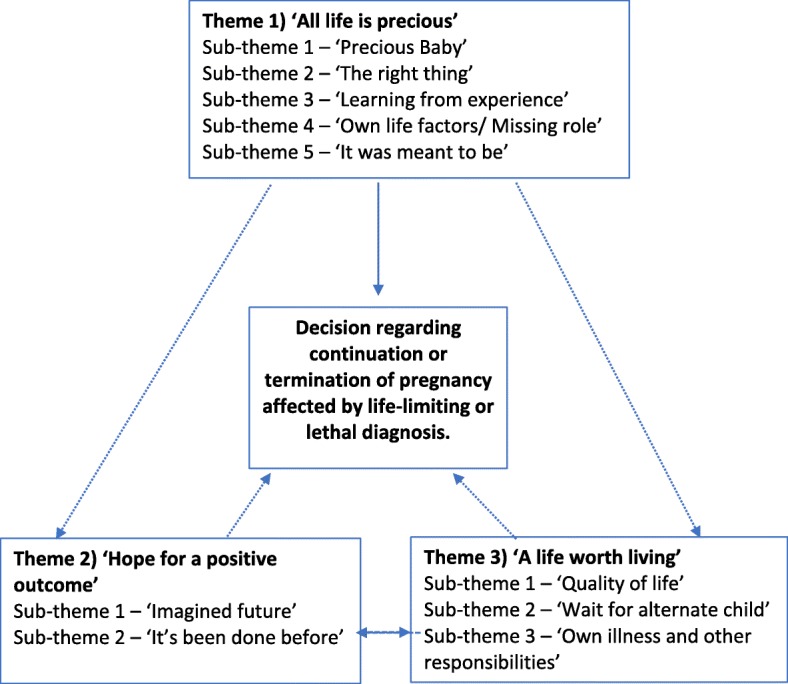
Diagrammatic representation of decision-making following lethal, life-limiting, or severely debilitating diagnosis

Three themes reflected aspects of influencing parental decision-making following a diagnosis of lethal, life-limiting, or severely debilitating condition: 1) ‘All life is precious’, 2) ‘Hope for a positive outcome’ and 3) ‘A life worth living’. These themes encompassed the main areas of decision-making for parents included within this review, with sub-themes present within each theme. The themes, their ten sub-themes, their relation to one another and the process of decision-making are depicted in Fig. 2. Quotes are used within the text to represent the themes.

#### Theme 1: all life is precious

A common theme throughout the studies was that parents viewed their fetus as precious: they considered their “baby’s worth” [47] and valued the potential life they might have (sub-theme 1). They also considered their own moral and religious beliefs and were guided into doing ‘The right thing’ (sub-theme 2) based on these judgements. Parents drew on their past-experience of pregnancy or terminations when considering whether they were able to continue or end a pregnancy, and described their views of life with disability and prior experience of this (sub-theme 3). Many studies reported that parents considered their own life pressures and circumstances which could have influenced their decision-making. They considered their beliefs about becoming a parent, with the anticipation or longing for the parent role (sub-theme 4). Finally, sub-theme 5 presented the fatalistic attitude parents described when considering the choices available to them, and whether they were able or fated to continue with a pregnancy that had “happened for a reason” [47].

##### Sub-theme 1: precious baby

Parents described how their connection with their unborn baby influenced their decision-making. They explained how processes such as seeing the baby’s face and fetal movement through ultrasounds had personified the baby, with the length of gestation adding to the sense of attachment. Another woman described her previous difficulties in conceiving, and as she had “tried so hard” [47] she was not able to consider ending the pregnancy. Often parents recognised that their attachment to their unborn baby influenced their decisions, and as a result they attempted to reduce the bond with the fetus, allowing them to then make a decision without influence from connection: “I was already 5 months pregnant with JayLynn so she was already a person to me” [54].

##### Sub-theme 2: the right thing

Almost all parents described wanting to act in an ethical manner when making decisions, based on moral and religious beliefs. Although the two domains of morality and religion overlapped at times, they appeared to be distinct entities for many parents, with one women referring to the decision being “a moral decision, not a religious one” [65], whilst another felt that her decision “had a lot to do with our religious faith” [47]. Parents described struggling with their conscience and beliefs around termination, with some women believing they would “go to hell” for terminating a pregnancy [58], and struggling with their decision until the process was over, believing that was “the last window of opportunity to stop it from happening” [62]. Some parents described seeking confirmation that their choice was morally acceptable by seeking advice from religious clergy. Often parents received the message that they should do “what they felt was necessary, even if it included termination” [46]; however, others were encouraged to continue with the pregnancy, with the message that “God would only give a person what they could cope with” [72].

##### Sub-theme 3: learning from experience

Parents considered learning from their own past experiences, reporting beliefs about the acceptability of termination which had “originated from past experiences with pregnancy termination” [47], with some stating it had influenced their current decision. Whilst parents’ contact with other people diagnosed with the disorder presented them with considerations of “all the good times” [77], they also discussed how they “remember the really hard times” [77], which added further intricacy to their decision-making.

“I already provoked an abortion once before, and now I think that it’s a punishment. I swore not to do it again” [64].

##### Sub-theme 4: own life factors

Parents discussed factors within their life circumstances which had influenced their choices around continuing or terminating an affected pregnancy. Parents highlighted prior attempts of becoming pregnant and suffering miscarriages or fertility difficulties, explaining that they had “tried so hard to have children” [47] and could not consider terminating the pregnancy. Some parents perceived that this pregnancy might be their last chance to have a child due to their advancing age, and felt that they had “waited long enough” [65] to conceive. However, other parents viewed increased age as a concern for their ability to care for an adult with life-limiting disorders when they themselves were older, with fears of “burdening other children or family with this child’s care after they died” [65].

##### Sub-theme 5: it was meant to be

Parents described a fatalistic attitude when deciding whether to continue or terminate pregnancies affected by lethal, life-limiting, or severely debilitating disorders, “believing that there was a reason or purpose for being pregnant with a child with Down syndrome” [53]. They explained that they believed there would be a higher purpose for any life, that they were fated to parent the affected child and that it was “meant to be” [54], placing value on the child they had been given, and accepting the outcome of the life regardless of difficulties.

“We were going to let Alaina determine the outcome of her life, no intervention in one way or the other” [54].

#### Theme 2: Hope for a positive outcome

The second theme identified was one of hope for a positive outcome. Parents described their hopes for the future, with a healthy or happy child. Parents explained how these hopes influenced their decision on continuing or terminating a pregnancy, based on whether they believed their imagined future was acceptable to them (sub-theme 1). Parents also described how their own and others’ belief in their ability to care for a child with lethal, life-limiting, or severely debilitating conditions influenced their decision to continue or terminate a pregnancy. Parents discussed how knowledge of other people’s experiences of parenting a child with similar disorders influenced their decision (sub-theme 2).

##### Sub-theme 1: imagined future

Parents explained how their hope that things would be manageable influenced their decision-making. They hoped that their child would survive, and that they and their wider family “would be able to spend some time” with their child [54]. Many parents hoped that all involved might experience some positivity from a traumatic situation.

Often parents did not have faith in the diagnostic certainty and questioned whether “aborting it or terminating it was really necessary” [66]. In these cases, they chose to conduct further research of their own, with the hope of finding positive outcomes for the disorders their unborn child had been diagnosed with or “verifying information from medical providers” [54]. Parents described finding “hope and comfort” [64] through information challenging the opinion of professionals, and many described requiring a “more trusted information source” [65] than professional opinion, instead seeking out “online support groups or blogs” [65] to further understand the disorder and make a decision.

“It was difficult, but you dust yourself off, you go home, you read up your books, you read the Internet, you know. And I think you’re able to then make informed choices.” [70]

##### Sub-theme 2: It’s been done before

The knowledge that other people had experienced similar situations as themselves was viewed as beneficial for decision-making. Seeking out “other people’s experiences” [72] and learning that other people valued their child, despite disabilities, with some hoping to “adopt a child with Down syndrome” [46], influenced some parents’ decisions to continue with a pregnancy. However, others were presented with facts from other parents within similar experiences, regarding the “really hard times that they had” [77] and encouraged to make a fully informed decision.

Parents’ beliefs in their own ability to “raise a child with Down syndrome” [77] or their own ability “to cope” [43] with the parental role influenced their decision-making. Many parents also relied on the belief others held about their ability to parent a child with specific difficulties, and found this influential in their decision-making process.

Advice given by others about different strains or severity of disorder influenced parental beliefs. Parents reported that children with the same condition, but a milder strain, were “going to school; it seems like they have no problems” [57]. Whilst parents were also reported to have harboured incorrect assumptions that “if the baby is diagnosed with thalassemia major in the first pregnancy, it will not re-occur in the second pregnancy” [57], leading to doubt in the accuracy of results and a choice to continue with their pregnancy.

One mother reported that when she called her sister with the news, her response was “You’d make a great mother for a kid with Down syndrome” [46].

#### Theme 3: a life worth living

The third theme was that of parents wanting to ensure their child’s life would be a life worth living. Parents’ decisions were influenced by their perception of what quality of life their child may encounter, both physically and emotionally, throughout their lives, as well as the impact caring for a child with difficulties would have on their own, and the quality of life of other members of their families (sub-theme 1). Some parents described their awareness that another child might not have these difficulties, and discussed their options of future healthy children as being influential in their decision-making process (sub-theme 2). Finally, a parent’s own illness and competing responsibilities influenced their decision around ability to continue with a pregnancy affected by lethal, life-limiting, or severely debilitating conditions, with parents considering the impact this would have on already stretched resources (sub-theme 3).

##### Sub-theme 1: quality of life

Parents considered the quality of life they anticipated their unborn child might have if they continued with a pregnancy, describing how this influenced their decision-making. Often parents reported that “they hoped that their child would remain comfortable throughout their life, without experiencing any pain or suffering” [54], considering the “likely extent and manageability of any physical or emotional suffering” [72]. Parents hoped that the child would be able to “feel or know that they were loved” [54], with some parents describing how they felt they could continue if “their baby could have a reasonable quality of life despite her/his condition” [72].

Other parents considered future implications on quality of life, and felt that they might find it “difficult to get treatment for this child” [77], with fears around how lack of treatment might impact on quality of life. Parents reflected on their child’s future life, and how it may not be what they would hope for themselves, if they had been given a choice.

Often parents considered the impact continuing or terminating a pregnancy would have on their own quality of life. For some parents, they imagined that continuing with a pregnancy would increase the value of their lives, believing that raising a child with a lethal, life-limiting, or severely debilitating condition would have brought them “closer together” as a family unit [54]. For other parents, the thought of the “emotional trauma” [65] of delivering and caring for a child who would eventually die was too much. Parents considered the risk to their “own health” [68] and “financial stability” [65], and how this might negatively impact their quality of life.

Parents also discussed the wider impact on wider family members’ quality of life, with particular reference to other siblings who could be affected by the child’s difficulties. Parents described how continuing with a pregnancy influenced their availability to support and guide their other children, considering the “added stress and burden” they could experience as a result of their unborn child’s specific needs and difficulties [77]. Parents also considered potential future ramification for siblings burdened “with this child’s care after they died” [65]. Some parents did describe how continuing with the pregnancy of a child with lethal, life-limiting, or severely debilitating disorders might add qualities to their other children’s life, and looked forward with excitement for their children “to experience this” [77]. They described how a child with specific difficulties would “bring to light a little less selfishness and a little more giving” [77] to their family unit, improving their overall quality of life.

“We came to the conclusion that the baby had a heartbeat, and the reason she had a heartbeat was because I was her lifeline. This was a baby on life support, basically, and I was her life support, and that was a turning point in our decision. If we’re in a horrible accident and a machine is pumping our blood and making us breathe and doing everything for us, we don’t want to live like that. If it’s good enough for us, why isn’t it good enough for this baby” [58]?

##### Sub-theme 2: wait for alternate child

Some parents anticipated future pregnancies and second chances at achieving the pregnancy they had hoped for, with an outcome they viewed as more favourable. Parents described wanting to “try again” [60] at creating a life which they had hoped for, and in turn achieve their “goals of having healthy children” [77].

For other parents, the association with birth and pregnancy as a joyful moment led them to consider whether the birth of a child with a lethal, life-limiting, or severely debilitating condition could change that. Some parents considered the impact this might have on their previous perception and “did not want to associate the beauty of delivery with suffering” [65].

##### Sub-theme 3: own illness and other responsibilities

Finally, parents also described how their own illnesses had led them to consider the impact of a child with life-limiting or lethal diagnosis, “because of the pressures of her own health condition” [72]. Similarly, some parents experienced their advanced age as a potential barrier to continuing with the pregnancy, describing how they were “thinking ahead” for the child’s needs in later life [77]. Parents also described how other life responsibilities and the perceived pressure of a child with lethal, life-limiting, or severely debilitating conditions on their family unit impacted their decision-making.

“Other events such as illness in the family or unsettled home life impacted some participants … and [at] that time our life was a bit of a mess because we’d sold the house and we were house sitting … it was pretty horrendous for that whole time … and still going on with the 10-month-old baby that we had and me being pregnant” [51].

## Discussion

This review, the first to focus solely on parental decision-making following prenatal diagnosis of a lethal, life-limiting, or severely debilitating conditions, augments our understanding of how parents arrive at a decision about their pregnancy. The review presents findings which support previous research suggesting that parents are influenced by values [17–21, 25], hope [25] and their own belief in their ability to care for a child with a lethal, life-limiting, or severely debilitating diagnosis [25]. A previous review highlighted the importance of prior experience or connection to others with experience of disability [25], an anticipation of the quality of life for themselves and their unborn child [25], and the perception of their child as precious [14, 25]. However, the current review also presents novel findings including those of the parents’ imagined futures influencing their decision-making. Unlike previous reviews which reflect on how parents prepare themselves for potential setbacks in future pregnancies, this review describes how parents also reflecting on their ability to conceive another child in the future and their own personal factors which may influence this, such as advancing age. Overall, the process of decision-making for parents following prenatal diagnosis was complex and personalised, with parents reporting different areas of influence on their own decisions. Parents described longed for pregnancies which influenced their decision-making, whilst other parents described an attachment to their fetus as being critical when forming a choice regarding pregnancy options. The majority of parents also placed a high value on their own view of the child as a precious lifeform, which coincided with moral and fatalistic beliefs and values.

Whilst none of the previous reviews considered decision-making models, one paper within this review drew on problem-solving decisional models when reflecting on parental decision-making following diagnosis of lethal, life-limiting, or severely debilitating disorders [79, 80] The role of decision-making theory is an important aspect to consider when attempting to understand parental experiences. The sense that all life is precious appeared to be the key influencer of parents faced with a decision to terminate or continue pregnancies. The individual and non-quantifiable construct of an individual’s belief in how worthwhile life is suggests that parents may well engage in more descriptive models of decision-making [29, 30], influenced by previous experiences and beliefs, rather than a more classical method of decision-making, whereby all potential outcomes are considered. Previous quantitative research had suggested that factors found within the main theme of ‘All life is precious’ are influential in parental decision-making, with the perceived fetus as precious [11] and attempts to do the right thing when making decisions [18–20]. The results from the current synthesis suggests that parents also consider personal life commitments, past experiences and fatalistic attitudes when considering whether to continue or terminate a pregnancy.

Whilst parental views of the importance of their fetus’ life appeared to be the main influencer of parents’ decisions, the current review also suggested that parents considered hope and the potential of the child’s life being worth living when making their decision. This coincides with findings from a previous review evaluating decision-making within affected pregnancies [25] which also described how hope for a successful outcome, alongside consideration of expected quality of life was an important consideration for parents.

It should be acknowledged that this review identified only studies from countries in which termination was a legal and viable option for parents. Furthermore, the removal of two low quality papers means that not all existing literature was included.

### Clinical and research implications

The current findings have important clinical implications for maternal healthcare teams supporting parents following diagnosis of lethal, life-limiting, or severely debilitating prenatal diagnosis as well as policy makers attempting to improve the overall care offered to parents during this time. Although this review highlights areas of shared importance for parents making these difficult decisions, it also emphasises the idiosyncratic nature of decision-making representative of descriptive models of decision-making [29, 30]. Professionals supporting parents should enable discussion around individualised thoughts and beliefs during the decision-making process and ensure that they do not anticipate rigid or prescriptive responses based on the choices of other parents with similar demographics. As advanced age was viewed as a precursor to continuation of pregnancy for some parents and a factor in termination for others, providing an open and supportive environment in which to enable parents to explore their options and the factors important to them would be beneficial.

Care teams should, however, be aware of parents’ beliefs around life, considering when they believe life begins, their attachment to the fetus, religious or moral values and any prior experiences which might have impacted their expectations of pregnancy or termination in order to support and scaffold parental decision-making and choice in a personalised and non-prescriptive manner.

Parents often struggled to accept the prognosis provided by medical professionals, doubting medical advice which did not facilitate hope, and wanting to conduct their own research to ensure there were no alternative pathways available to them. They also reported finding the experience of others helpful in alerting them to both hope of a life worth living, and the realistic difficulties they would face if continuing with their pregnancy.

Other parents considered the quality of life for their child and family unit when making their choice. Support could be provided by care teams facilitating contact with relevant groups and other parents who could provide insight into their own lived experience as a way of parents exploring this important factor themselves.

A tiered model of psychological care could be facilitated for parents following diagnosis of lethal, life-limiting, or severely debilitating disorders. The provision of tiered psychological care has been widely utilised within physical health care settings [81–83], with a multi-disciplinary team approach to providing psychological support to individuals experiencing emotional suffering. This could be provided through ward staff, and for a smaller number of parents struggling with adjustment or decision-making, through the provision of psychological practitioners such as Clinical Psychologists, in line with current recommendations for psychological care [84].

## Conclusion

Whilst it has long been accepted that a parent’s decision-making between termination or continuation of a pregnancy identified with a lethal, life-limiting, or severely debilitating condition is an emotionally traumatic and challenging experience [10], the specific influencers of decision-making were yet to be explored thoroughly. Given the consistency of themes found across all 22 included studies despite geographical differences, the findings appear to be applicable across different maternal healthcare systems in various countries. The current review highlights the importance of parents’ perception of the fetus’ life, basing their decision on moral beliefs and past-experience. It also suggests that some parents considered hope and the impact on their own and the fetus’ life when making their decisions. Increasing the understanding of factors influencing parental decision-making will enable maternal healthcare professionals to provide an avenue for the exploration of these factors within a supportive environment and improve current services offered to parents following diagnosis.

## Data Availability

All data generated or analysed during this study are included in this published article.
